# Ex vivo resection and intestinal autotransplantation for a large mesenteric desmoid tumor secondary to familial adenomatous polyposis: A case report and literature review

**DOI:** 10.1097/MD.0000000000010762

**Published:** 2018-05-18

**Authors:** Chao Cheng, Shuang Guo, Dakinah Eastman G. B. Kollie, Wanli Zhang, Jun Xiao, Jun Liu, Xiaoming Lu, Yong Xiao

**Affiliations:** a Department of Gastrointestinal Surgery; b Department of Pathology; c Cancer Center, Union Hospital, Tongji Medical College, Huazhong University of Science and Technology, Wuhan, Hubei Province, China.

**Keywords:** ex vivo resection, FAP, feasible technique, intestinal autotransplantation, mesenteric desmoid tumor

## Abstract

**Rationale:**

The mesenteric desmoid tumor requires special attention and the most demanding treatment.

**Patient concerns:**

Here we present a rare case of a large mesenteric desmoid tumor secondary to familial adenomatous polyposis (FAP) in a 34-year-old man accepted the ex vivo resection, and intestinal autotransplantation.

**Diagnoses:**

A 34-year-old man was referred to our department with a 6-year history of intermittent hematochezia without any other discomfort after undergoing partial colectomy in February 2013, and 5 endoscopic mucosal resections of colon polyps between May 2012 and July 2015 due to pathological diagnosis of FAP. A computed tomography scan showed a huge abdominal mass with indistinct boundary at the root of the mesentery. The adjacent organs were pushed and most of the superior mesenteric artery branches were infiltrated.

**Interventions:**

An en bloc resection (R0 resection), and an ex vivo resection followed by intestinal autotransplantation was performed.

**Outcomes:**

The patient was discharged from the hospital on the 25th day after the operation, and was regularly followed up after surgery with abdominal ultrasonography and laboratory-biochemical tests every month, and serial CT scans every 3 months which showed no evidence of tumor recurrence, thrombus, intestinal obstruction or abdominal infection so far.

**Lessons:**

An ex vivo resection and intestinal autotransplantation appear feasible for cases with pathological lesions involving the vessels at the root of mesentry, and represents an attractive alternative for the management of mesenteric desmoid tumors.

## 1 Introduction

Desmoid tumor is the most common primary tumor of mesenteric fibromatoses, and tends to have a particularly high incidence in patients with familial adenomatous polyposis (FAP), especially after surgery.^[1]^ Despite its benign cytology, desmoid tumor shows aggressive features such as infiltration of surrounding tissue which makes it a potential life-threatening condition which requires special attention.^[2]^ Once the large and vital vessels of the mesentery are involved by desmoid tumor, complete resection is impossible by using conventional techniques which may result in uncontrollable bleeding and subsequent short bowel syndrome. Recently, small-bowel autotransplantation for tumor involving the mesenteric root has prevailed against specific technical obstacles; however, this surgical procedure was just performed on limited patients and recorded in few researches.^[3]^ Here we report a successful tumor extirpation with free surgical margins, and segmental intestinal autotransplantation of a large mesenteric desmoid tumor secondary to FAP.

## 2 Case presentation

The patient provided informed consent for the publication. The study was approved by the ethics institutional review board of the Union Hospital of Huazhong University of Science and Technology.

**Figure 1:**
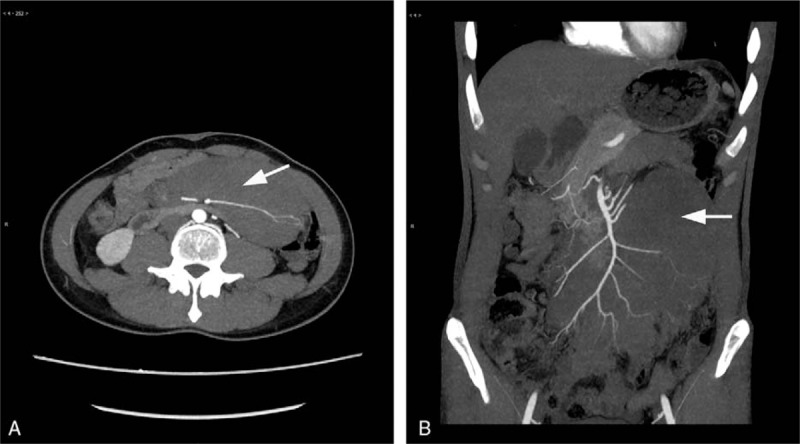
Preoperative computed tomography (CT) shows tumor extension. Representative images of transverse section (A) and coronal section (B). The arrow indicated the lesion. CT = computed tomography.

A 34-year-old man presented a 6-year history of intermittent hematochezia without any other discomfort. He underwent partial colectomy in February 2013, and 5 endoscopic mucosal resections of colon polyps between May 2012 and July 2015, respectively. Postoperative pathological analysis showed mixed hyperplasia adenomatous polyps of sigmoid colon, and was finally diagnosed as FAP. An abdominal mass was palpated in June of 2017, and ultrasonography confirms a solid tumor. Needle biopsy showed a few spindle cells with enlarged and dense nucleus, but malignant tumor could not be excluded. Further examination by computed tomography scans demonstrated a large soft tissue mass with indistinct boundary at the root of the mesentery (Fig. 1, arrow). The adjacent organs were pushed and most of the superior mesenteric artery branches were infiltrated (Fig. 1).

The patient did however present in an excellent general condition, well nourished despite the weight loss, and extremely motivated for an aggressive surgical treatment of the tumor. No other comorbidity factors contraindicated the operation. Based on referencing other surgeons’ reports, our experience, and small-bowel autotransplantation experimental researches on animals, we concluded that he was a perfect candidate for small bowel segment autotransplantation. The ex vivo resection and intestinal autotransplantation was performed in Wuhan Union Hospital on September 18, 2017.

**Figure 2:**
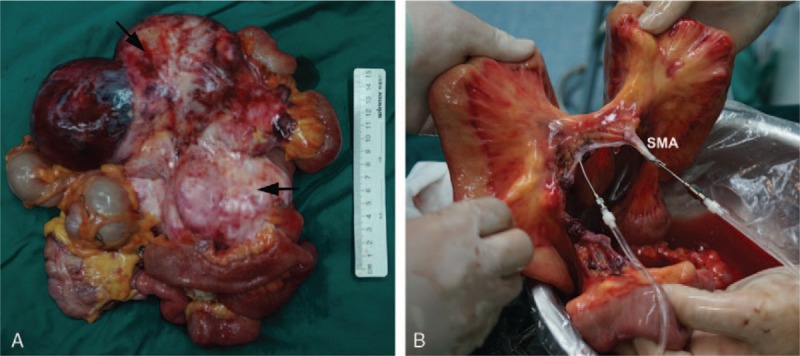
An en bloc resection was performed (A) and the specimen was flushed through the SMA with cold preservation solution to protect isolated small bowel (B). The arrows indicated the lesion. SMA = superior mesenteric artery.

Upon entering the abdominal cavity through a midline incision, we found that the tumor was approximately 20 cm × 15 cm, originating from the mesentery root, and adhered tightly to the descending part of the duodenum, a portion of small intestine and the head of the pancreas. The superior mesenteric artery (SMA) and the superior mesenteric vein (SMV) were engulfed by the tumor. An en bloc resection was performed, including the whole small intestine, right and proximal transverse colon, while the SMA and SMV were resected at their root. The surgical specimen was removed and arranged to an ice salver on a separate specimen table, and flushed through the SMA with cold University of Wisconsin preservation solution (ViaSpan, Barr Laboratories Inc., Pomona, NY) to protect isolated small bowel (Fig. 2). About 150 cm jejunum and its mesenteric vascular arch were completely stripped from the tumor ex vivo. And then, they were reimplanted to enterocoelia, while the vascular and the digestive tract were reconstructed. The proximal jejunum was anastomosed to the duodenum and the distal part was pulled out from the abdominal wall for a jejunostomy.

**Figure 3:**
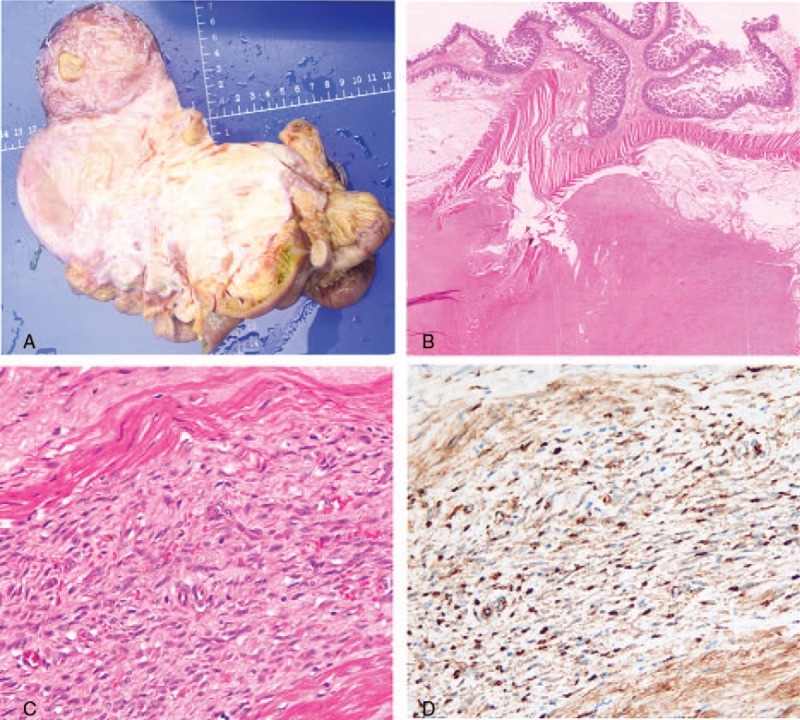
Pathologic assessment of the tumor. (A) The cut surface showed a trabecular and myxoid appearance. (B) The small intestinal wall was infiltrated. (C) The high magnification vision showed bland cytologic features. (D) The β-catenin stained strongly at the nuclear by immunohistochemical staining.

Postoperative pathologic examination showed a gritty, and poor circumscribe neoplasm originating from the small intestine mesentery which tightly adhered to the surrounding organs. A cross-section revealed a glistening white, coarsely trabeculated surface, and myxoid appearance in some area (Fig. 3A). Microscopically, the lesion infiltrated the small intestine and colon wall (Fig. 3B). Bland spindle-shaped cells composed the tumor cytologically (Fig. 3C). Immunohistochemical staining showed strong nuclear β-catenin staining (Fig. 3D). These findings supported a diagnosis of mesenteric desmoid tumor. All resection margins were negative, which demonstrated a complete surgical excision (R0).

The borborygmus gradually returned to normal on postoperative day 2, and bile-like digestive juices from the jejunostomy was observed on postoperative day 3. At the 10th day after surgery, the jejunostomy was ruddy, and the patient weaned off parenteral nutrition and started to take fluid for enteral nutrition. When the patient tended to be in a stable condition, he was discharged from the hospital on the 25th day after the operation with the discharge medication of anticoagulants, multi-vitamins and enteral nutrition suspensions. The patient was regularly followed up every month after surgery with abdominal ultrasonography and laboratory-biochemical tests every month, and serial CT scans every 3 months which showed no evidence of tumor recurrence, thrombus, intestinal obstruction or abdominal infection so far.

## 3 Discussion

FAP syndrome is caused by a germline mutation of the APC gene. Palmirotta et al^[4]^ demonstrated that germline and somatic inactivation of the APC gene is the fundamental step in the onset of desmoid tumors, and the intranuclear accumulation of β-catenin protein caused by the mutation of the APC gene can be detected by using immunohistochemistry as a diagnostic adjunct. As described previously, approximately 10% to 15% of patients with FAP develop intra-abdominal fibromatosis.^[5–7]^ Furthermore, mesenteric desmoid tumors which generally occur after resection of the pathological lesion portion of the intestinal tract is the most common cause of death in polyposis patients who underwent prophylactic colectomy.^[8]^ Histologically, the tumors present with a benign nature that composed of elongated, spindle cells evenly deposited in a densely collagenous or myxoid stroma with rare mitotic figures.^[9]^ However, they can cause significant morbidity and mortality due to their characteristics of infiltrating the surrounding structures.^[10]^

Although operative resection is regarded as the main treatment for symptomatic desmoid tumors, unfortunately, conventional resection can be complicated by excessive bleeding and ischemic damage to the intestine or a need for massive enterectomy.^[11]^ Recently, partial exenteration, ex vivo resection, and intestinal autotransplantation surgery has rendered to solve these obstacles in resection of such neoplasms.^[3,12–14]^ The physiologic base of this technique is using a cold solution (University of Wisconsin solution) to create a prolong time of intestinal cold ischemia status, avert irreversible damage and allow surgeon to resect the tumor with subsequent intestinal autotransplantation.^[3,15]^ This technique has several advantages, such as excellent exposure of surgical field, optimum control of the critical step of tumor resection in a bloodless field, clear visualization of vascular pedicles, and arches to protect normal tissues or ensure accurate excision, and so on. Nevertheless, this procedure remains technically challenging because of its complexity which includes the involvement of multiple organs, prolonged operative time, and increased blood transfusions. Thus, only few case reports have described patients with mesenteric tumor encasing the blood supply of the small intestine, ex vivo resection, and subsequent intestinal autotransplantation.^[3,16,17]^

In this case report, we successfully performed such surgical procedure on a FAP patient who developed a large mesenteric desmoid tumor. The large mesenteric desmoid tumor was extirpated with free surgical margins, and about 150 cm jejunum with its mesenteric artery and vein branches was reimplanted into enterocoelia after cold flushing ex vivo to restore normal intestinal continuity. Different from other reports, we resolved to choose a distal jejunostomy instead of enterocolostomy to conveniently observe the status of the small intestine. So far, the patient recovered smoothly after surgery.

There are few researches setting focus on the indications of ex vivo resection and subsequent intestinal autotransplantation. Whether pathological lesions involving the vessels at the root of mesentery is suitable for palliative treatment or curative excision remains controversial. We consider that patients with high-grade malignancy may not benefit from this technique, whereas low-grade especially insensitive to chemoradiotherapy ones, may obtain curative excision. For this case, it has been confirmed that the large soft tissue mass with indistinct boundary at the root of the mesentery pushing against the adjacent organs infiltrated most of the superior mesenteric artery branches as proven by ultrasonography and CT scans, respectively. Needle biopsy showed a few spindle cells with enlarged and dense nucleus despite the potential risk of malignancy. However, the patient presented in excellent general condition, well nourished, and extremely motivated for an aggressive surgical treatment to the tumor, and no other comorbidity factors contraindicated the operation. Therefore, the ex vivo resection and intestinal autotransplantation was performed.

According to our clinical experiences combined with experiments on animals, we can conclude that: First of all, preoperative assessment must be rigorous. The tumor size, infiltrate condition, and encase degree of the vessels at the root of mesentery should be explicit by MRI, CTA or other necessary clinical examinations. Pathologic inspection on biopsy should provide a specific result as possible. For those lesions that extensively infringe the small bowel, and cause insufficient intestine to satisfy intestinal nutrition, this technique may have no effect, and also this new operation procedure is not suitable for patients with high-grade malignancy. After excluding surgical contraindications, surgeons should adopt effective measures to improve the nutritional status of the chosen patients to tolerate the surgery.

Next, the superior mesentery vessels and well-vascularized small intestine must be preserved carefully for the sequence autotransplant during the operation. A direct anastomosis for vascular reconstruction is recommended to avoid the potential risks of infections and keep long-term vascular potency. We preferably choose small intestine ostomy to observe the survival status, and to avoid anastomotic leakage which is more likely to occur in this kind of patients due to the lack of anastomotic blood supply that might be a fatal complication to them. The borborygmus and the color, character and amount of excreta from the stoma reflects the recovery status of intestinal function. Based on the postoperative recovery status of patients, the small intestinal stoma closure operation could be performed 4–6 months after the jejunostomy.

Furthermore, antibiotics and intravenous heparin should be routinely given to prevent infections and thrombogenesis after the surgery.

In conclusion, ex vivo resection and intestinal autotransplantation seems to be a feasible technique for patients with pathological lesions involving the vessels at the root of mesentry. Careful preoperative assessment, refined operation, and meticulous postoperative care are the keys to the success of this aggressive and complicated surgery. Our experience may support the use of this technique as a potential option for the treatment of patients with a large mesenteric desmoids tumor which cannot be radically removed by conventional surgery.

## Author contributions

**Methodology:** Xiaoming Lu.

**Project administration:** Xiaoming Lu.

**Resources:** Jun Liu.

**Software:** D. Eastman G.B. Kollie.

**Supervision:** Jun Liu, Yong Xiao.

**Visualization:** Jun Xiao.

**Writing – original draft:** Shuang Guo, D. Eastman G.B. Kollie, Wanli Zhang.

**Writing – review & editing:** Chao Cheng.
